# Deciphering the interactions between single arm dislocation sources and coherent twin boundary in nickel bi-crystal

**DOI:** 10.1038/s41467-021-21296-z

**Published:** 2021-02-11

**Authors:** Vahid Samaee, Maxime Dupraz, Thomas Pardoen, Helena Van Swygenhoven, Dominique Schryvers, Hosni Idrissi

**Affiliations:** 1 Electron Microscopy for Materials Science (EMAT), University of Antwerp, Antwerp, Belgium; 2 Photons for Engineering and Manufacturing, Paul Scherrer Institut, Villigen PSI, Switzerland; 3 IRIG MEM NRS, CEA Grenoble, Grenoble, France; 4 XNP, ESRF, Grenoble, France; 5 Institute of Mechanics, Materials and Civil Engineering, UCLouvain, Louvain-la-Neuve, Belgium; 6 Neutrons and X-Rays for Mechanics of Materials, Ecole Polytechnique Fédérale de Lausanne, Lausanne, Switzerland

**Keywords:** Mechanical properties, Microscopy

## Abstract

The introduction of a well-controlled population of coherent twin boundaries (CTBs) is an attractive route to improve the strength ductility product in face centered cubic (FCC) metals. However, the elementary mechanisms controlling the interaction between single arm dislocation sources (SASs), often present in nanotwinned FCC metals, and CTB are still not well understood. Here, quantitative in-situ transmission electron microscopy (TEM) observations of these mechanisms under tensile loading are performed on submicron Ni bi-crystal. We report that the absorption of curved screw dislocations at the CTB leads to the formation of constriction nodes connecting pairs of twinning dislocations at the CTB plane in agreement with large scale 3D atomistic simulations. The coordinated motion of the twinning dislocation pairs due to the presence of the nodes leads to a unique CTB sliding mechanism, which plays an important role in initiating the fracture process at a CTB ledge. TEM observations of the interactions between non-screw dislocations and the CTB highlight the importance of the synergy between the repulsive force of the CTB and the back stress from SASs when the interactions occur in small volumes.

## Abstract (web summary)

Interactions of dislocations with coherent twin boundaries contribute to strength and ductility in metals, but investigating the interaction mechanisms is challenging. Here the authors unravel these mechanisms through quantitative in-situ transmission electron microscopy observations in nickel bi-crystal samples under tensile loading.

## Introduction

The interaction of dislocations with coherent twin boundaries (CTBs) of the type ∑3 {111}〈110〉 has been extensively studied since the early observations of twinning in face centred cubic (FCC) metals^1^. The demonstration that the toughness (i.e. strength ductility product) of a poly-crystalline FCC metal can be enhanced when promoting a high density of grown-in twins^2,3^ suggests that the CTB acts as a barrier for dislocation slip (increasing the strength) while allowing under certain conditions, slip transfer and the generation of new dislocations (contributing to enhancing the ductility). This discovery motivated a realm of researches to study the interaction of dislocations with CTBs using dedicated experiments inside electron microscopes or with atomistic simulations. However, despite intensive research, the elementary processes that control the reaction mechanism(s) at the CTBs are still subject to debate^4,5^. Indeed, multiple parameters such as the stacking fault energy (SFE), the type, length, and curvature of the incoming dislocation, the image force of the CTB and the loading conditions could play a role in whether a dislocation gets absorbed or transmitted and how transmission occurs^6–9^. On the other hand, performing dedicated quantitative in-situ characterizations of the dislocation/CTB interactions still constitutes a major experimental challenge. In the literature, both ex-situ transmission electron microscopy (TEM) and conventional in-situ TEM deformation experiments have been used to investigate the interaction of dislocations with CTBs^6–8,10,11^. However, these studies suffer from the absence of quantitative information on the interaction mechanisms due to the lack of force-sensing capabilities with classical in-situ TEM deformation holders. More recently, new advances in in-situ nanomechanical testing methods have enabled more quantitative characterizations based on focused ion beam (FIB) prepared bi-crystalline micro and nanopillars with single a ∑3 {111}〈110〉 CTB inside a scanning or transmission electron microscope^12–18^. However, although micro-tension is generally used to overcome most of the experimental shortcomings of the micropillar compression approach such as the deformation of the substrate and the lateral constraint between the pillar top surface and the compression tip, quantitative in-situ TEM tensile testing on FIB defect-free bi-crystal samples is still missing in the literature. One problem is that FIB-induced defects could significantly affect the dislocation/CTB interactions mechanisms.

**Fig. 1:**
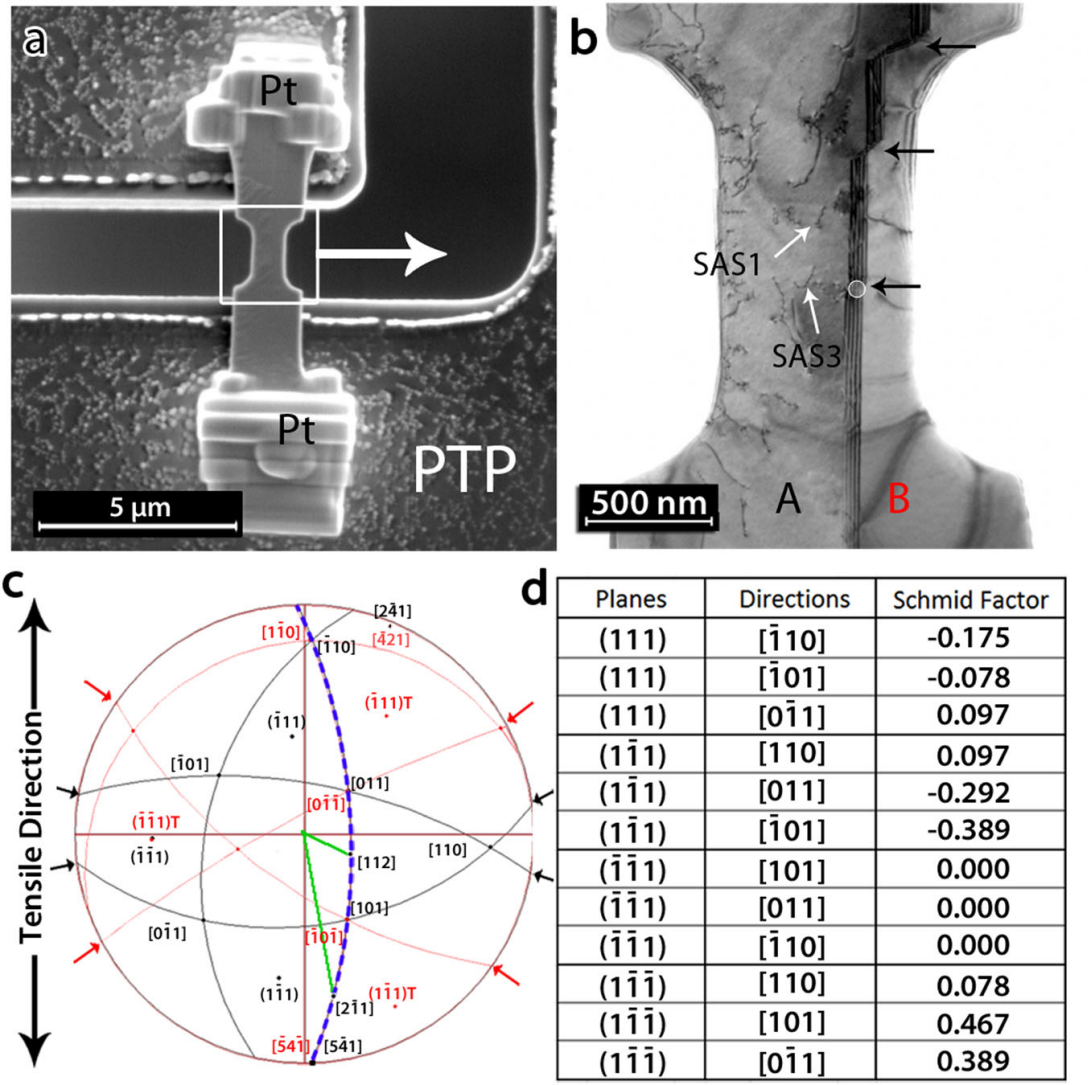
Sample preparation for quantitative in-situ TEM tensile testing. **a** SEM micrograph showing the sample mounted on a Push-to-Pull (PTP) device, see more information in the “Methods” section and the supplementary information. **b** Bright field TEM micrograph of the sample before deformation (white square and arrow in **a**). The white circle indicates the location of a TB ledge used as a reference in Fig. 2. Single arm sources SAS1 and SAS2 are indicated by white arrows while the black arrows show the position of ledges at the CTB. **c** Overlapped stereographic projections of grains A (black) and B (red). The TB plane $(\bar 1\bar 11)$ or $(\bar 1\bar 11)_T$, is marked by a blue dashed line. **d** Schmid factor values of the slip systems in both grains. The two slip planes with the highest Schmid factor, $(1\bar 1\bar 1)$ and $(1\bar 11)$, are marked with black and red arrows, respectively, in **c**.

In this work, we perform on a pure Ni bi-crystal sample quantitative in-situ TEM observations under uniaxial tensile testing of the interaction mechanisms between curved screw dislocations with a single ∑3 {111}〈110〉 annealing CTB parallel to the tensile axis. The Ni bi-crystal sample was prepared by FIB from electropolished 3 mm discs containing several annealing CTBs following the procedure described by Samaee et al.^19^. In order to avoid FIB-induced damage, the electropolished sample was heat treated using in-situ annealing in the TEM. In order to facilitate the nucleation of dislocations, which is often a major concern in such small-sized specimens^19^, the time and temperature of the heat treatment were intentionally selected to keep only a few dislocations within the in-situ TEM tensile sample. EDX measurements have shown that the amount of Ga left after annealing is below the EDX method accuracy of 1 at.%^19^. The commercial PI95 PicoIndenter holder (Brucker Inc.) and a dedicated silicon Push-to-Pull (PTP) device were used to deform the specimen^19,20^ (see more details in the “Methods” section and Supplementary note 1). Figure 1a shows the Ni bi-crystal sample after mounting on the PTP device using Pt source inside the SEM chamber. Figure 1b shows a bright field TEM (BF-TEM) image obtained from the bi-crystal sample prior to straining. The ∑3 {111}〈110〉 CTB exhibits a few ledges indicated by black arrows. Dislocations that survived the heat treatment can also be observed. The stereographic projections of the two grains A and B are shown in Fig. 1c. Since the tensile axis is (almost) parallel to the CTB plane (blue dashed line in Fig. 1c), the tensile direction in both grains is close to the $5\bar 41$ direction and the Schmid factors of the slip systems are similar in both grains (see Fig. 1d). Black and red arrows in Fig. 1c indicate the two slip planes with the highest Schmid factors in grains A and B, respectively.

## Results

### Transition from transmission to absorption of screw dislocations

**Fig. 2:**
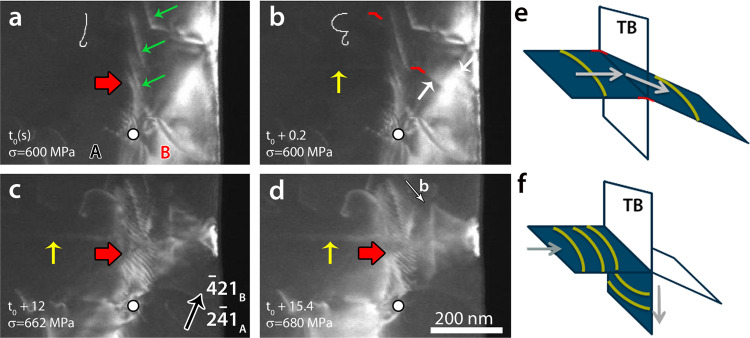
Transition from transmission to absorption of screw dislocations. Snapshots from the Supplementary movie 1 of cycle 2, showing the dynamic of SAS1 **a** just before activation, and **b**–**d** at different times during deformation. The yellow (resp. white) arrows indicate the freshly formed ST in grain A (resp. grain B). Red segments in **b** are used to indicate the connection between STs in both grains (see also the schematic in **e**). Note that the Burgers vector of the dislocation of the SAS is parallel to the intersection between the gliding plane of the dislocation and the CTB plane. Red arrows show the formation of fringe-like contrast at the CTB indicating the absorption of the incoming dislocations at the CTB (see the schematic in **f**). The white dot shows the location of the TB ledge used as a reference.

**Fig. 3:**
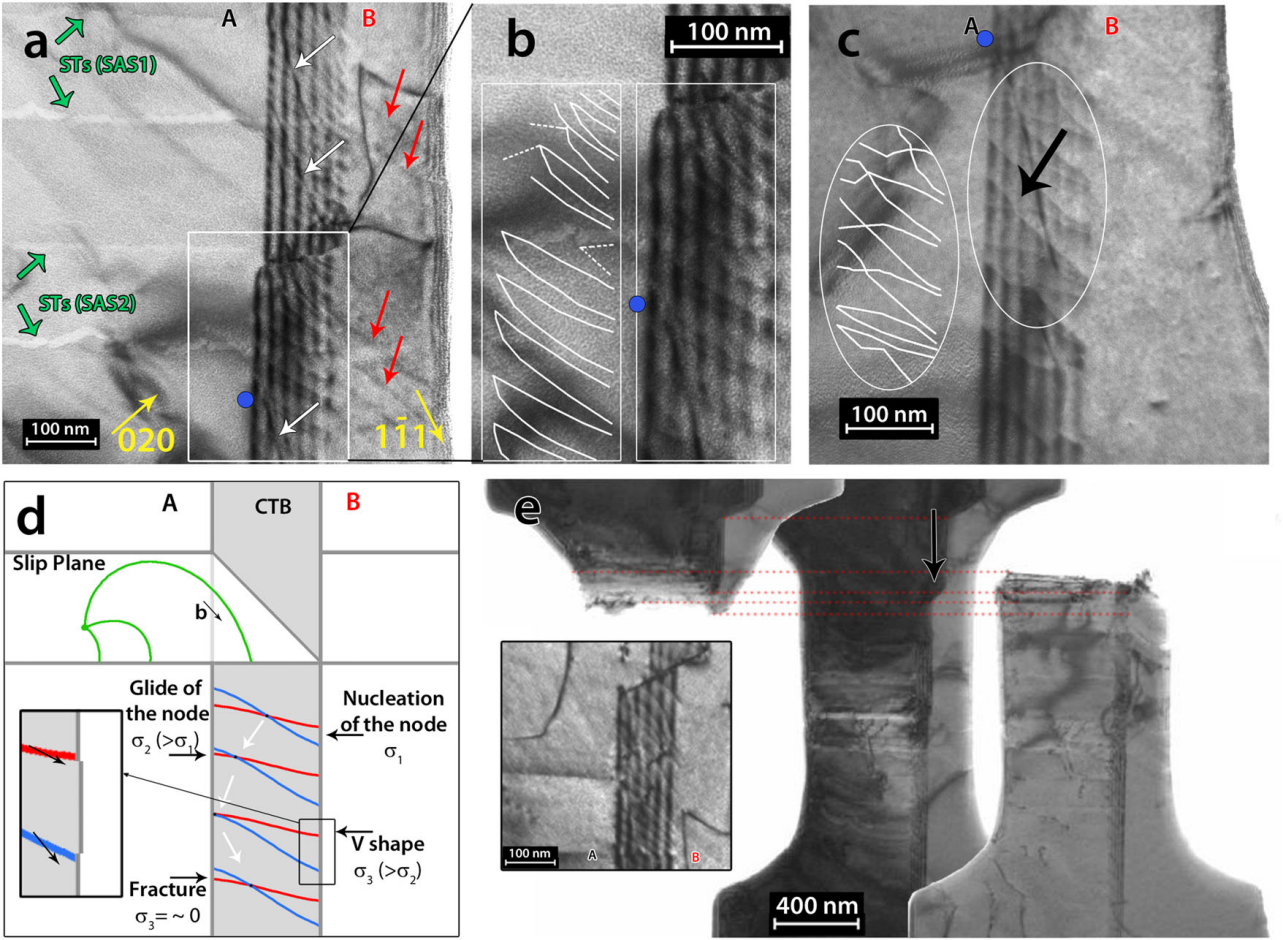
Mechanism of absorption of screw dislocations at CTB. **a** and **b** Bright field TEM micrograph after cycle 4 showing the arrangement of the twinning dislocations resulting from the absorption process of the incoming screw dislocations at the CTB. Green arrows indicate the position of STs from the single arm sources SAS1 and SAS2. Yellow arrows indicate the diffraction vectors while red arrows show the position of slip traces in grain B after transmission. White arrows indicate residual sessile dislocations left by annealing. White lines in **b** show the arrangement of the twinning dislocations in the white rectangle. **c** Bright field TEM image obtained after fracture. The black arrow show the position of a constriction node. White lines show the arrangement of the twinning dislocations. **d** Schematic illustration of the mechanism controlling the absorption process of the incoming curved screw dislocations (green lines): it involves the nucleation and the glide of constriction nodes in the same direction at the CTB plane as well as the backward motion of the nodes by relaxation of internal stress in the CTB after fracture. The two TDs of Eq. (1) form a pair of up-down single atomic steps; see the red and blue lines inside the black rectangle. **e** BF-TEM image showing the fracture at the CTB ledge indicated by a black arrow. The inset shows the accumulation of twinning dislocations (resulting from the absorption process) at the CTB ledge.

The sample shown in Fig. 1 was subjected to five loading–unloading cycles with maximum applied engineering stress of (1) 550 ± 13 MPa, (2) 693 ± 17 MPa, (3) 703 ± 17 MPa, (4) 793 ± 20 MPa, and finally (5) 1084 ± 27 MPa at which fracture occurred. Except for the second cycle, the sample was kept under maximum load (i.e., load plateau) for 1 min. In cycles 2 and 4, two single arm sources (SASs) have been activated. In Fig. 2, the yellow arrows indicate a slip trace (ST) resulting from the activation of SAS1 (Fig. 1b) in the $(1\bar 1\bar 1)$ plane of grain A during cycle 2 at a stress of 600 ± 15 MPa; see Supplementary notes 2 and 3 for more details on the determination on slip planes and Burgers vectors of SASs. Note the enhancement of the contrast of this ST with increasing stress (Fig. 2b–d) due to the continuous operation of SAS1. In Fig. 2b, at *t* = *t*_0_ + 0.2 s after the activation of SAS1, slip transfer was observed as evidenced by the appearance of STs in grain B (white arrow in Fig. 2b), which are connected to the STs from SAS1 in grain A (see also Supplementary note 4). No dislocation pile-up against the CTB was detected here. At 662 ± 16 MPa and *t* = *t*_0_ + 12 s (Fig. 2c), a transition from slip transmission to absorption was observed. This is evidenced by the sudden increase of the number of dislocations moving in a direction parallel to the CTB plane (red arrows in Fig. 2c and d); see also Supplementary movie 1. A similar behaviour was observed in cycle 4 for the SAS2 activated at 793 ± 17 MPa in grain A; see Supplementary note 5 and Supplementary movie 2. The BF-TEM image of Fig. 3a obtained after cycle 4 shows the STs made by the operation of SAS1 and SAS2 (green arrows) as well as STs originating from slip transfer to grain B from these sources (red arrows). Note that SAS2 was identified using the STs. The exact position of this source is not indicated in Fig. 1b because its activation was too fast to be captured in a single frame. The width of the ST bands in grain A indicates that the slip planes are $\left( {\overline 1 11} \right)$ while systematic contrast analysis of SAS1 confirmed that the Burgers vector is $a/2\left[ {101} \right]$ which is parallel to the CTB plane (see Fig. 2d and Supplementary notes 2 and 3). A similar Burgers vector is expected for SAS2 since the STs originating from this source are very similar to those of SAS1 (see Supplementary note 3). The observations shown in Figs. 2 and 3 thus involve the interaction between screw dislocations and a ∑3 {111}〈110〉 CTB; see extra evidence on the screw character of the incoming dislocations in Supplementary note 4.

The slip transmission that occurred immediately after the activation of the SASs could be explained by the orientation of the CTB with almost zero Schmid factor in the CTB plane. The fast transmission without the formation of dislocation pile-ups against the CTB^8,17,21^ can be attributed to the high resolved shear stress (RSS) required to activate short sources in submicron specimens (in comparison with bulk samples). In this work, the RSSs at which slip transmission of screw dislocations was observed (280 ± 7 MPa for SAS1 and 370 ± 9 MPa for SAS2) are in a good agreement with the estimated specific threshold RSS required for screw dislocation transmission in Ni reported by Jin at al. using MD simulations (300 ± 7–380 ± 9 MPa)^5^. In high SFE metals such as Ni, atomistic simulations have shown that the Friedel-Escaig mechanism occurs explained by the fact that it requires low activation energy^2,4,5,22–24^. This mechanism involves the constriction under the applied stress of a pair of leading and trailing partial dislocations to form a perfect dislocation interacting with the boundary. The transition from slip transmission to absorption with increasing stress shown in Fig. 2 can be explained by the increase of the RSS in the TB plane due to small misalignment during the mounting of the specimen on the PTP device and/or local changes of orientation resulting from the geometrical constraints imposed by the PTP mounting setup. Indeed, the sample is constrained on both sides, which can result in the destruction of the symmetry and in the creation of bending and rotation moments in addition to the uniaxial force when a large slip event preferentially occurs on a single set of planes. Such behaviour is an interesting finding since it can be compared to other situations in which non-uniaxial stresses as well as stress conditions including shear components acting in the CTB could appear^24^ and increase upon deformation such as in twinned polycrystalline materials.

### Mechanism of absorption of screw dislocations at CTB

The incoming screw dislocations in Fig. 2c could cross-slip on a slip plane parallel to the TB plane^4^ or get absorbed at the CTB by cross-slip and dissociation inside the CTB plane into two twinning dislocations (TDs) following the reaction:

1 $$a/2\left[ {101} \right]_{(\bar 111){\mathrm{{Matrix}}}} = a/6\left[ {112} \right]_{(\bar 1\bar 11){\mathrm{{TB}}}} + a/6\left[ {2\bar 11} \right]_{(\bar 1\bar 11){\mathrm{{TB}}}}$$

In the BF-TEM images of Fig. 3a and b taken after loading cycle 4, the dislocations resulting from the absorption of the incoming screw dislocations are arranged in pairs in agreement with the cross-slip and dissociation mechanism of Eq. (1). Furthermore, despite the limitations imposed by the absence of the double-tilt option in the PicoIndenter holder, systematic contrast analysis using single tilt showed that the dislocations at the TB could be either $a/6\left[ {112} \right]$ or $a/6\left[ {2\bar 11} \right]$ TDs in agreement with the dislocations involved in Eq. (1) (see also Supplementary note 6). Interestingly, most of the dislocation pairs in Fig. 3b are forming a V shape configuration covering the entire CTB plane. The coupling between the TDs of Eq. (1) confirms that the interaction between screw dislocations and a ∑3 {111} CTB leads to CTB sliding instead of CTB migration. Indeed, the two TDs of Eq. (1) form a pair of up-down single atomic steps that induce CTB sliding when moving in the same direction^5,22,25–27^. The glide of the same two TDs in opposite directions would induce CTB migration by one atomic step^25^. However, one important question remains at this stage: what is the mechanism that keeps the TDs of Eq. (1) paired in Fig. 3b?

Based on 3D concurrent atomistic continuum (CAC) simulations, Xu et al. reported a transition from CTB migration to CTB sliding with increasing RSS during interaction of screw dislocations with a ∑3 {111} CTB in Al. However, the origin of such behaviour was not elucidated^22^. Furthermore, to the best of the author’s knowledge, quantitative experimental evidences have not been provided yet. In the BF-TEM image of Fig. 3c, after the fracture of the sample, some paired TDs forming V shapes can still be observed. However, several constriction nodes connecting the two TDs of Eq. (1) are seen at the CTB plane. These constriction points are the result of the interaction process between the incoming screw dislocations and the CTB as will be demonstrated later. Very recently, Dupraz et al. used large 3D-MD simulations with free boundary conditions to investigate the interaction mechanism between a screw dislocation and a ∑3 {111} CTB under uniaxial tensile loading in pure metals^24^. The results revealed the role of the dislocation curvature that cannot be captured in 2D-MD simulations. More precisely, Dupraz et al. demonstrated that, when a curved dislocation with a dominant screw character interacts with a CTB, there is a competition between transmission and absorption leading to the formation of a constriction node between the two TDs of Eq. (1) in the CTB plane. Such behaviour occurs when the RSS on the incoming dislocation is below the critical stress required for slip transmission. In the present work, similar 3D-MD simulations are performed to investigate the formation of a constriction node by the interaction of curved screw dislocations with a CTB in Ni, not reported in the work of Dupraz et al.^24^. Indeed, because the screw dislocations are nucleated from SASs very close to the CTB (Fig. 2), curved screw dislocations interact with the boundary.

**Fig. 4:**
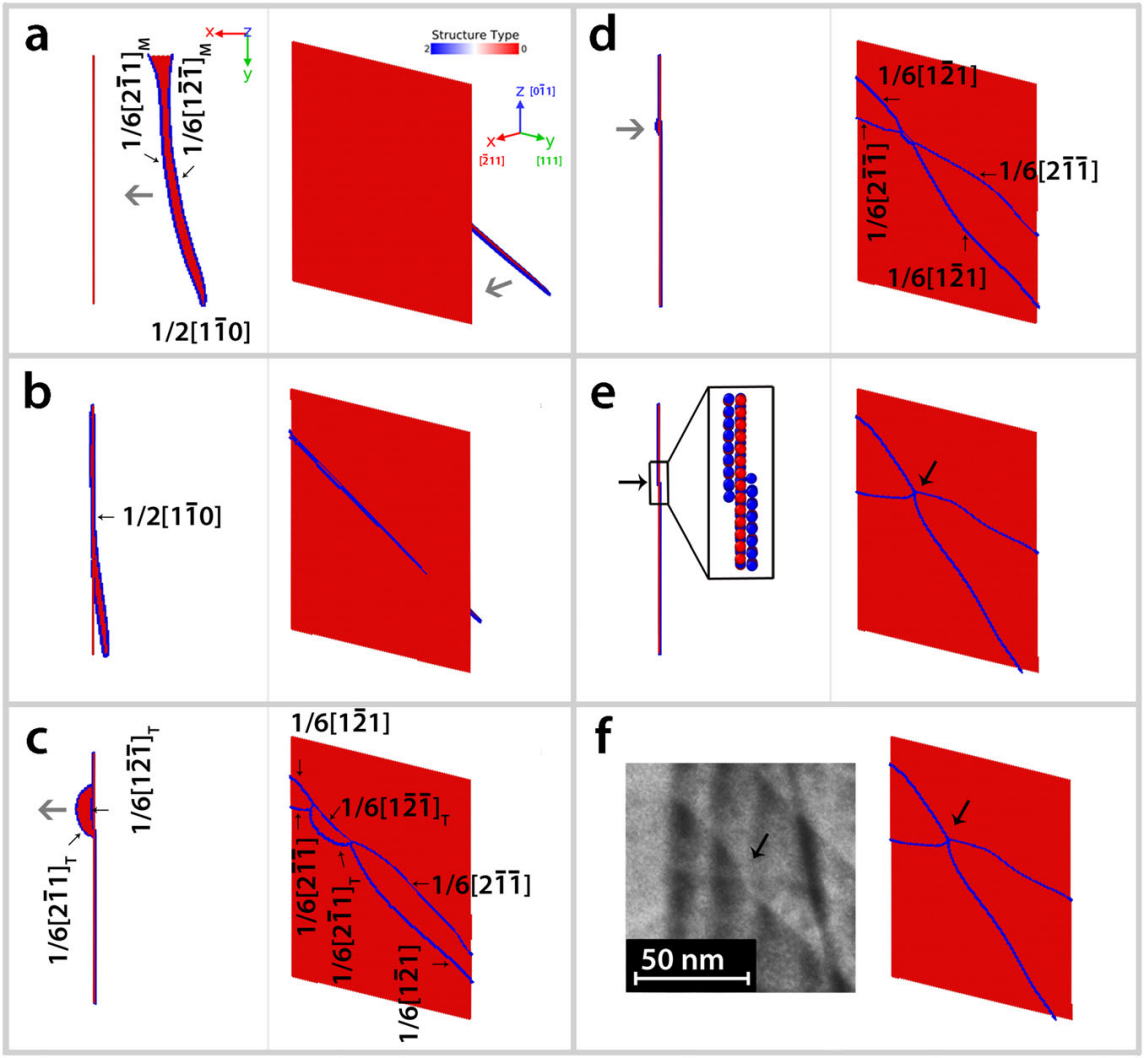
large 3D-MD simulations of the formation mechanism of the constriction node in the CTB. Sequence of snapshots showing the formation of the constriction node in the CTB under uniaxial Z-tension (supplementary movie 4). The resolved shear stress acting on the screw component ${\mathbf{\sigma }}_{{\boldsymbol{rss}}\_\_{\boldsymbol{screw}}}$ is equal to 204 ± 5 MPa and below the critical stress for transmission. **a** Dissociated screw dislocation propagating in the incoming grain. **b** Constriction into a perfect screw dislocation in contact with the CTB. **c** Competition between absorption in the$\left( {111} \right)$ CTB plane and transmission on the $\left( {\overline 1 11} \right)$ plane of the twinned grain. **d** and **e** Reabsorption of the cross-slip nucleus in the twinned grain resulting in the formation of a construction node. The magnified image in the black rectangle in **e** shows that the constriction node is a TB step of three atomic planes with edges located on both sides of the CTB. **f** Comparison between the simulated node in **e** and the experimentally observed node indicated by black arrow in Fig. 3c. The atoms are coloured according to the CNA analysis, only the non-FCC atoms are shown.

Figure 4 shows the sequence of events in the MD simulations leading to the formation of the constriction node in the CTB plane (see also Supplementary note 8 and Supplementary movie 4). Here, the loading is applied parallel to the CTB plane along the $[0\bar 11]$ crystallographic direction (Fig. 4a). This configuration is very similar to the present experiment where the loading direction is almost parallel to the CTB. Under such loading conditions, the curved dislocation with dominant screw character experiences a large RSS (Schmid factor = 0.408). It starts gliding towards the CTB once the RSS counterbalances the repulsive force from the CTB as well as the image forces from the surface^23^. The interaction mechanism starts only after the constriction of the two Shockley partials into a full dislocation with ***b*** = $\frac{a}{2}[1\bar 10]$ (Fig. 4b). As the incoming screw dislocation interacts with the CTB, cross-slip nuclei are observed simultaneously in CTB plane ($\frac{a}{6}\left[ {2\bar 1\bar 1} \right]$ and $\frac{a}{6}\left[ {1\bar 21} \right]$ partials) and in the adjacent twinned grain ($\frac{a}{6}\left[ {2\bar 11} \right]$ and $\frac{a}{6}\left[ {1\bar 2\bar 1} \right]$ partials) (see Fig. 4c). As shown in ref. ^24^, above the critical transmission stress the cross-slip nucleus in the twinned grain grows at the expense of the cross-slip nucleus in the CTB, enabling the transmission of the dislocation across the CTB. However, below the critical transmission stress, the cross-slip nucleus in the twinned grain dissolves in favour of the cross-slip nucleus in the CTB, resulting in the formation of a constriction node (Fig. 4d, e). In Fig. 4f, one can note the similarity between the simulated node in Fig. 4e and one of the experimentally observed nodes indicated by a black arrow in Fig. 3c. To the best of our knowledge, The TEM observations shown here thus provide first of a kind experimental evidences of the constriction nodes resulting from the competition between transmission and absorption of curved screw dislocations with a CTB. Priester et al.^11^ reported dislocation nodes within complex networks of interfacial dislocations in ∑3 {111} CTB in Ni bi-crystal after annealing in-situ inside the TEM. However, the formation of such nodes involves complex reactions between CTB dislocations from different sources where the climb of dislocations could play an important role.

Because the presence of a constriction node keeps the two TDs coupled during their glide within the CTB, the following scenario (Fig. 3d) is proposed to explain the mechanisms involved in the absorption process leading to the TDs arrangement shown in Fig. 3. First, the interaction between the curved screw dislocations and the CTB leads to the formation of the constriction nodes as demonstrated in the 3D-MD simulations of Fig. 4. Because of the identical nature of the nodes, they move by unzipping in the same direction driven by the external applied stress (Fig. 3d). However, in contrast with MD simulations where the constriction point could easily escape to the free surface with zero RSS in the TB plane, Fig. 3b shows that the nodes cannot escape to the surface in the in-situ TEM experiment, leading to the formation of the V-shaped pairs of TDs observed in the same figure. The origin of such feature can be attributed to the larger size of the sample compared to MD simulations as well as to the elastic interactions within the CTB that can lead to the equilibrium configuration shown in Fig. 3b. Note that further 3D-MD simulations have shown that increasing the size of the simulation cell significantly increases the lifetime of the constriction node (see Supplementary note 9 as well as Supplementary movies 4–6). Furthermore, Fig. 4e shows that the constriction node is a TB step of three atomic planes with edges located on both sides of the CTB. This geometry might also play a role in the stabilization of the node. After fracture, the stress in the CTB plane is relaxed resulting in the backward motion of several nodes from the surface to the CTB interior (Fig. 3c and d). It is worth noting that, the movement of the absorbed dislocations observed in Supplementary movie 1 indicates that, in addition to their lateral glide by unzipping, the constriction nodes could also vertically glide parallel to the CTB leading to CTB sliding.

Figure 3e shows that fracture occurred at the CTB ledge as indicated by a black arrow in the same figure in cycle 5 at 940 ± 23 MPa. This can be attributed to the accumulation of TDs of Eq. (1) at the CTB ledge (see Fig. 2c, d, the inset in Fig. 3e and Supplementary movie 1) leading to large stress concentrations. Emission of dislocations from the TB ledges was not observed. The presence of the constriction nodes and the associated CTB sliding mechanism could accelerate the fracture process at the CTB ledge since higher number of TDs could accumulate at this ledge. CTB migration process involving the glide of the two TDs of Eq. (1) in opposite direction (without constriction nodes) would however induce changes of the height of the CTB ledge. It is also worth noting that few residual sessile dislocations left by annealing have been observed at the CTB; see green arrows in Fig. 2a and white arrows in Fig. 3a. These dislocations might affect the dynamics of the absorbed TDs but not the intrinsic absorption mechanism as supported here by large 3D-MD simulations.

### Interaction between CTB and non-screw dislocations

**Fig. 5:**
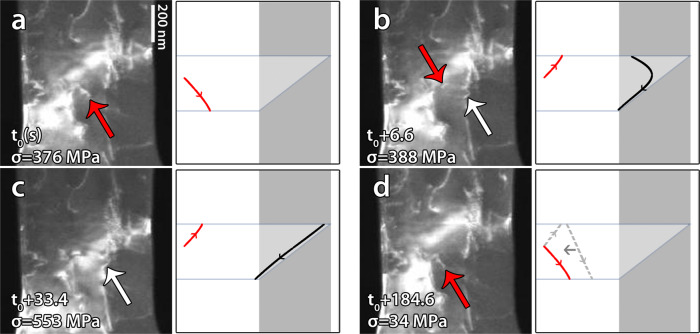
Interaction between CTB and non-screw dislocations. **a**–**c** Snapshots from Supplementary movie 3 of cycle 3 showing the microstructure and the corresponding schematics observed during the interaction between non-screw dislocation from the single arm source SAS3 and the CTB at different times. Red (resp. white) arrows indicate the position of the source (resp. the incoming dislocation). Note the reflection of the dislocation at the CTB followed by the annihilation of a segment of the same dislocation with the source leading to the final configuration shown in (**d**).

In cycle 3, SAS3 (Fig. 1b) has been activated under a stress of 376 ± 9 MPa (Fig. 5a). Using the width of the ST bands as well as systematic contrast analysis, the dislocation’s slip system is $a/2\left[ {\bar 101} \right]\left( {1\overline 1 1} \right)$ with Burgers vectors inclined with respect to the CTB plane (see Supplementary note 7 for more details). In Fig. 5b, a dislocation that was formed from this source has been blocked at the CTB at 388 ± 9 MPa (see also Supplementary movie 3). Note the curved character of this dislocation at the CTB with one segment aligned parallel to the intersection between the glide plane of the incoming dislocation and the CTB plane while the other segment remains inclined with respect to this intersection. In Fig. 5c, the entire dislocation line was forced to align parallel to the intersection at 553 ± 13 MPa. During unloading, Fig. 5d shows a backward motion of this dislocation from the CTB to SAS3. This confirms that the dislocation did not enter the CTB even at such a high stress level. When the dislocation approaches the source, annihilation between segments of opposite signs occurred at 34 ± 1 MPa leading to the configuration shown in the micrograph of Fig. 5d (see also the schematic illustration in the same figure). Such feature can be attributed to a synergy between the repulsive image force of the CTB and the attractive force applied by the source on the dislocation. Atomistic simulations have shown that the dislocation/CTB interaction is repulsive at large distances^4,23^. At short distances, depending on the material, the interaction can be attractive such as in Al^5^ or repulsive such as in Cu and Ni^4^.

In summary, quantitative in-situ TEM tensile testing of Ni bi-crystal specimen was performed in order to investigate the elementary mechanisms controlling the interaction between dislocations and a ∑3 {111} CTB parallel to the uniaxial tensile direction. The results show fast transition from slip transmission to dislocation absorption during interaction of curved screw dislocations nucleated from SASs with the CTB. The absorption process leads to the formation of constriction nodes connecting pairs of dissociated TDs in the CTB plane, in perfect agreement with large 3D-MD simulations. The presence of the nodes keeps the dissociated dislocations paired during their glide leading to a CTB sliding mechanism. Fracture occurred at a CTB ledge due to the accumulation of the dissociated paired dislocations at the ledge and the associated CTB sliding mechanism. Reflection of a non-screw dislocation at the CTB was observed at high stress levels followed by the annihilation of the dislocation with the SAS, which highlights the importance of the synergy between the repulsive force of the CTB and the back stress from SASs when the interactions occur in small volumes. Our findings provide precious local information that can be used in multiscale modelling approaches to guide the optimization of the mechanical properties of heavily twinned face-centred cubic metals with nanoscale CTBs in materials design. The experimental approach used in the present work opens avenues to investigate the mechanisms controlling the interaction between dislocations and other classes of structural boundaries in a quantitative manner.

## Methods

### Specimen preparation

First, a high purity Ni foil (99.999%) (Goodfellow GmbH, Bad Nauheim, Germany) was annealed for 1 h at 400 °C and then punched to 3 mm discs. After grinding and mechanical polishing, the discs were electropolished with a solution of perchloric acid and acetic acid, 1:4, in a Tenupol 3 instrument (Struers ApS, Ballerup, Denmark) at 0 °C, 18–19 V, and 100 mA. The electro-polished 3 mm discs were then investigated by TEM in a Tecnai G2 microscope (FEI Company, Hillsboro, OR, USA) operating at 200 kV in order to select proper regions in terms of thickness and location/direction of annealing CTBs. A FEI Helios Nanolab 650 dual beam FIB/SEM was then used to produce the dog bone shape tensile samples by the Ga^+^ ion beam (30 kV/80 pA) at the preselected locations (see Supplementary information). In order to avoid FIB-induced damages, the electropolished sample was then heat treated using a Gatan in-situ TEM heating holder for ~1 h at 700 °C^19^. In order to facilitate the nucleation of dislocations, which is often a major concern is such small-sized specimens^19^, the time and temperature of the heat treatment were intentionally chosen to keep a few dislocations within the in-situ TEM tensile sample. FIB equipped with an Omniprobe (Oxford Instruments plc, Tubney Woods, UK) was used after the in-situ TEM heat treatment in order to mount the tensile specimen on the PTP device using Pt deposition (Fig. 1a).

### In-situ TEM nanomechanical testing

The commercial PI95 PicoIndenter TEM holder (Brucker Inc.) and a dedicated silicon PTP device were used to perform quantitative in-situ TEM tensile testing^19,20^ (see also supplementary information). The in-situ TEM tensile experiments were carried out in the load-control mode with a loading rate of 1 nN/s (initial strain rate of $\sim 7 \times 10^{ - 5}\frac{1}{{\mathrm{s}}}$) in a FEI Osiris TEM operating at 200 kV. The length of the reduced section of the PTP specimen was 1 μm, the width 800 nm and the thickness was around 200 nm. Overlapped diffraction spots $2\bar 41$ (grain A) and $\bar 421_T$ (grain B) from the two grains were used for dark field imaging during in-situ experiments.

### 3D-MD simulations

Interactions between Ni atoms were modelled with the Mishin potential^28^. This potential was selected because it predicts a SFE in good agreement with the experiment. The simulation cell consists of two grains that are mirrored across the $\left( {\bar 1\bar 11} \right)$ plane. This common mirror plane represents the twin boundary. Free surfaces were considered as boundary conditions. The lattice orientations corresponding with the axes of the simulation cell are *X*$[111]$, *Y*$[\bar 211]$, *Z*$[0\bar 11]$ and *X*′$\left[ {111} \right]_T$, *Y*′$[2\bar 1\bar 1]_T$, *Z*′$[01\bar 1]_T$ for the incoming and outgoing (twinned) grains, respectively. In directions *X*, *Y*, and *Z* the cell lengths are *Lx* = $108a\sqrt 3$, *Ly* = $40a\sqrt 6$ and *Lz* = $70a\sqrt 2$, corresponding to ~7.2 million atoms. After an initial relaxation of the system with energy minimization, a perfect screw dislocation with ***b*** = $\frac{a}{2}[1\bar 10]$ was introduced on the $(\bar 1\bar 11)$ plane, ~150 Å away from the CTB (Fig. 1b), using the free software Atomsk^29^. The equilibrium configuration of the system obtained after energy minimization consists of two Shockley partials:

2 $$\frac{1}{2}\left[ {1\bar 10} \right] = \frac{1}{6}\left[ {1\bar 2\bar 1} \right] + \frac{1}{6}\left[ {2\bar 11} \right]$$

MD simulations were carried out with the open-source large-scale atomic/molecular massively parallel simulator (LAMMPS)^30^ using a Nose–Hoover thermostat in the NVT ensemble. The temperature was set to 10 K. The uniaxial tensile loading was performed along the $[0\bar 11]$ crystallographic direction (*Z*-direction, see Fig. 4). As illustrated in Fig. 4, the CTB plane is parallel to the loading direction therefore there is no shear stress in the boundary plane. The loading was applied in stress-controlled mode by applying traction forces to the atoms in 12 Å wide slabs (larger than the cut-off radius of the potential). Under such loading conditions the dislocation experiences a high RSS (Schmid factor of 0.408) driving it towards the boundary. The stress was applied in a single increment by prescribing opposite sign forces on the slabs at the positive and negative *Z* free surfaces. In order to minimize the stress fluctuations, the system is given an initial deformation which takes into account both the elastic and thermal expansions.

## Supplementary information

Supplementary Information

Description of Additional Supplementary Files

Supplementary Movie 1

Supplementary Movie 2

Supplementary Movie 3

Supplementary Movie 4

Supplementary Movie 5

Supplementary Movie 6

## Data Availability

The datasets generated during and/or analysed during the current study are available from the corresponding author on reasonable request.
