# Supplementary material for: Deciphering the interactions between single arm dislocation sources and coherent twin boundary in nickel bi-crystal
Source: Nat Commun. 2021 Feb 11;12:962. doi: 10.1038/s41467-021-21296-z (PMC7878869; doi:10.1038/s41467-021-21296-z)
Supplement: Supplementary file 1 — Supplementary Information [file 41467_2021_21296_MOESM1_ESM.pdf]

Supplementary Information

Deciphering the interactions between single arm dislocation sources and coherent twin boundary in nickel bi-crystal

Vahid Samaee[1], Maxime Dupraz[2,3,4], Thomas Pardoen[5], Helena Van Swygenhoven[2,6], Dominique Schryvers[1] & Hosni Idrissi[5,1]*

[1] Electron Microscopy for Materials Science (EMAT), University of Antwerp, Groenenborgerlaan 171, B-2020 Antwerp, Belgium.

[2] Photons for Engineering and Manufacturing, Paul Scherrer Institut, 5232 Villigen PSI, Switzerland.

[3] IRIG MEM NRS, CEA Grenoble, 17 Avenue des Martyrs, Grenoble, 38000, France

[4] XNP, ESRF, 71 Avenue des Martyrs, Grenoble, 38000, France

[5] Institute of Mechanics, Materials and Civil Engineering, UCLouvain, B-1348, Louvain-la-Neuve, Belgium.

[6] Neutrons and X-Rays for Mechanics of Materials, Ecole Polytechnique Fédérale de Lausanne, 1015 Lausanne, Switzerland.

*The corresponding author: hosni.idrissi@uclouvain.be

Supplementary note 1. Specimen preparation and in-situ TEM tensile testing

After electro-polishing, the 3 mm discs were investigated using conventional TEM in order to select proper regions in terms of thickness, electron transparency, and orientation of the CTB. FIB milling of dog-bone shape specimens was thus achieved on selected annealing CTBs (Supplementary Figure 1a). Spring-like features were designed at the end of each tensile specimen to damp the probable stress induced by thermal expansion during heat-treatment inside the TEM. After in-situ TEM heating for ~1 hour at 700°C, a bi-crystal dog-bone specimen with almost zero FIB damage was mounted on the push-to-pull (PTP) device (Supplementary Figure 1b) in a dual beam FIB/SEM workstation equipped with an Omniprobe and a GIS. Supplementary Figure 1c shows the final configuration of the specimen after mounting on the PTP device. Special attention was paid not to expose the gage part of the dog-bone specimen to the Ga ion beam during the FIB mounting step.

With its four springs at the corners (Supplementary Figure 1b), the PTP device enables converting an external applied compression load to a tension load on the specimen mounted on the small gap marked by white square in Supplementary Figure 1b. In the present work, a PTP with stiffness of $150\ \mathrm{N}/\mathrm{m}$ was used in the PI 95 PicoIndenter holder. The quantitative in-situ TEM tensile tests were performed using the load control mode in a FEI Osiris Tecnai TEM. The specimen was subjected to 5 loading-unloading cycles with maximum engineering stresses equal to 550 ± 13 MPa, 693 ± 17 MPa, 703 ± 17 MPa, 793 ± 20 MPa and 1084 ± 27 MPa. Failure of the specimen occurred in the last cycle at stress level of 940 ± 23 MPa. In each cycle, the specimen was kept under the maximum load for 1 minute (load plateau). Supplementary Figure 2 exhibits an example of the evolution of load with time in cycle 3 up to 703 ± 17 MPa. In cycle 2, due to a technical problem, the specimen was unloaded after reaching the maximum load without any load plateau. The TEM-dark field (DF) mode was used in most deformation cycles to facilitate the observation of dislocation/CTB interaction mechanisms. However, due to the very low diffraction contrast in this mode, digital image cross-correlation of the movies was inaccurate. Therefore, full engineering stress-strain curves are not provided.

The actual load applied to the sample was obtained by subtracting the spring contribution from the raw load data. The engineering stress (Eng-stress) was extracted by estimating the initial cross-section area of the gage of the sample. This was achieved by measuring the width

and the thickness of the sample from a TEM image. The extraction of the thickness was achieved by measuring the width of the projection of a known active slip plan (144 nm in Supplementary Figure 3(a) for SAS1 activated in the $(\bar{1}11)$ slip plane). Thus, knowing the angle between this slip plane and the surface of the sample (46° in Supplementary Figure 3(b)), the thickness of the sample can be estimated as 207 nm. The uncertainty on the applied stress is mainly due to the precision of the measurement of the width and the thickness of the tensile sample from the TEM images (± 5 nm) as well as that of the force applied on the sample (± 0.2 μN). According to the propagation rules, a standard error on the cross-sectional area of ± 4000 nm^2 and on each engineering stress (given in "Results") was obtained.

Supplementary note 2. Identification of the slip planes

The crystallographic orientation of a pair of slip traces (STs) as well as the distance between the two STs in TEM images are used to identify the corresponding active slip plane. For instance, in grain B, the STs of slip planes $(1\bar{1}\bar{1})$ and $(1\bar{1}1)$ can be easily discriminated (red lines in Figure 1c). When the orientations of two different pairs of STs are rather identical, the distance between STs for each pair is used. Indeed, the distance between the paired STs in TEM images depends on the angle between the slip plane and the specimen surface, i.e., when the angle is high (close to 90°), the distance between two STs is short and when the angle is low, the distance is long. Supplementary Figure 4 shows the STs of $(\bar{1}\,1\,1)$ and $(1\,\bar{1}\,1)$ planes in grain A. The distance between the STs in Supplementary Figure 4a and Supplementary Figure 4b is shorter than the ones in Supplementary Figure 4c indicating that the slip plane in Supplementary Figure 4a and Supplementary Figure 4b is $(1\,\bar{1}\,1)$ while the slip plane in Supplementary Figure 4c is $(\bar{1}\,1\,1)$.

Supplementary note 3. Characterization of single arm sources

Although the single tilt nature of the pico-indenter holder impedes the use of systematic contrast analysis to fully characterize the dislocations, it can still be used to discriminate between dislocations. Supplementary Figure 5 shows two-beam TEM-BF micrographs taken with diffraction vectors $g = 220$ and $g = 200$ in grain A. According to Supplementary Figure 5 and Supplementary Table 1, the Burgers vector of visible dislocations cannot be $a/2\,[0\bar{1}1]$. Among the $a/2\,[110]$ and $a/2\,[101]$ dislocations, the Burgers vector of SAS1 activated in the

$(\bar{1}11)$ plane is likely $^{a}/_{2}\,[101]$ because the Schmid factor (0.467) is higher compared to $^{a}/_{2}\,[110]$ dislocations (0.078). Furthermore, detailed analysis of the STs formed at the surface of the sample by SAS1 and SAS2 (Supplementary Figure 6a) clearly show that the shear displacement is parallel to $[101]$; see Supplementary Figure 6b. Such observation is made possible by tracking at the STs roughness features revealed by the presence of Pt nanoparticles at the surface. Pt inevitably re-sputters on the surface in the form of nanoparticles during the mounting of the sample on the PTP. These particles are visible in the insets of Supplementary Figure 6a.

Supplementary note 4. Cycle 2

We compared the length of SAS1 measured by TEM with the one calculated from the equation below used for sources in small grains or thin films. It is modified from the known equation for a Frank-Read source by replacing the length L of the source by L/2 [1–3].

Equation S1 $$CRSS = \frac{\alpha G\boldsymbol{b}}{4\pi l}\left(\ln\left(\frac{2l}{b}\right) + 1\right)$$

where α is a coefficient equal to 1 or 1/(1-ν) for edge or screw dislocations, respectively; G is the shear modulus (76 GPa); b is the magnitude of the Burgers vector (0.249nm), ν is the Poisson's ratio (0.31) and L is the SAS length. A good agreement was found between the length of SAS1 measured by TEM (57 nm) and the source length extracted from the previous equation (55.3 nm) for a pure screw SAS activated at a CRSS of 600 MPa. Because the theoretical calculations match very well with the experimental results without the need for including a contribution from a Cottrell atmosphere, the effect of residual Ga atoms which might be present at the core of the SASs on the activation of these sources cannot be considered as a dominant mechanism.

Supplementary Figure 7 shows the transmission of dislocations from SAS1 through the CTB. In this figure, ST(A)2, ST(B)1 and ST(B)2 can be easily recognized. The contrast of ST(A)1 is, however, much weaker due to the local increase of the deviation parameter (s) but can still be identified by the intersection between the rotating arm of SAS1 and the surface (red arrow in Supplementary Figure 7a). In Supplementary Figure 7b, four red lines are drawn to confirm the connection between the STs from both sides of the CTB. The slip traces intersect at the two vertical blue lines showing the intersection between the CTB plane (defined by the CTB dislocations) and the free surface. The transmission of the screw dislocations can also be

clearly observed in the Supplementary movie 1 with the simultaneous formation of these four slip traces immediately after the activation of SAS1. Supplementary Figure 8 is a snapshot from the Supplementary movie 1 showing that the dislocation segment interacting with the CTB has a dominant screw character (white arrow in the right edge). Similar segment (but with opposite sign) can be observed at the left edge of grain A, confirming the screw character of the incoming dislocations.

Supplementary note 5. Cycle 4

In cycle 4, SAS2 starts operating in grain A at a stress of 793 ± 20 MPa in the load plateau (red arrows in Supplementary Figure 9a and 9b show the formation of STs in grain A). This was rapidly followed by slip transmission in grain B (new STs indicated by blue arrow in Supplementary Figure 9b). Then, similarly to the scenario observed for SAS1 in cycle 2 (in the main text), a transition from slip transmission to absorption was observed as evidenced by the glide of new dislocations parallel to the CTB plane (see white arrows and the inset in Supplementary Figure 9c and 9d). After a short time, SAS2 shutdown in the same load plateau. The identical relative shear displacements at the STs of SAS1 and SAS2 (Supplementary Figure 6) indicate that the Burgers vectors of SAS1 and SAS2 are the same.

Supplementary note 6. Characterization of CTB dislocations

Supplementary Figure 10 shows two-beam images of the dislocations absorbed at the CTB. The visible dislocations in Supplementary Figure 10a vanish in Supplementary Figure 10b indicating that the *g.b* product with $g = 020$ is ~ 0. According to Supplementary table 2, these dislocations might be ${}^{a}/_{6}\,[112]$ and ${}^{a}/_{6}\,[2\bar{1}1]$ in agreement with the cross-slip dissociation mechanism of Equation (1) in the main text.

Supplementary note 7. Interaction of a non-screw dislocation with the CTB

In the loading part of cycles 3 and 4, a dislocation nucleated from SAS3 (Supplementary Figure 5) interacts with the CTB (Figure 5 in the main text and Supplementary movie 3). A comparison between the length of SAS3 before and after the activation of this source shows that the length of SAS3 decreased leading to the shutdown of the source. Using the width of the pair of STs, the slip plane was determined as $(1\,\bar{1}\,1)$. The visibility of SAS3 (Supplementary Figure

5 and Supplementary Table 3) confirms that the Burgers vector of the dislocation is either $^{a}/_{2}\,[\bar{1}\;0\;1]$ or $^{a}/_{2}\,[1\;1\;0]$, neither of which is parallel to the CTB plane. It can thus be anticipated based on Schmid factor analysis that the Burgers vector is $^{a}/_{2}\,[\bar{1}\;0\;1]$. This is in agreement with the screw character of the segment that remains inclined with respect to the intersection between the glide plane of the incoming dislocation and the CTB plane (see Supplementary Figure 5b and Supplementary Figure 11). Indeed, since the line tension of a screw dislocation is smaller than of an edge dislocation, a freely moving dislocation tends to align itself and to glide with a dominant screw character in order to minimize its energy.

Supplementary note 8. Burgers vector determination of the TB dislocations in 3D-MD simulations

Supplementary Figure 12 shows the Burgers vectors of the twin boundary dislocations resulting from the absorption of a curved screw dislocation after formation of the constriction node. The analysis is carried out using the dislocation analysis (DXA) package implemented in ovito[4]. The analysis confirms that the order of the partials is inverted on either side of the constriction node (Supplementary Figure 12b). Note that the absorbed segment on the left side of the constriction node is formed in the vicinity of a free surface, which could assist the inversion of the order of the partials, allowing the formation of the constriction node.

Supplementary note 9. Size effect on the stability of the constriction node

In order to quantitatively evaluate possible size effects on the stability of the constriction node, additional simulations with different sizes of the simulation cell were performed. Two additional simulation cells were considered: a small one with Lx_{small} = ½*Lx_{medium}, Ly_{small} = ½*Ly_{medium} and Lz_{small} = ½*Lz_{medium}, and a large one with Lx_{large} = Lx_{medium}, Ly_{large} = 3/2*Ly_{medium} and Lz_{large} = 2*Lz_{medium}. Lx_{medium} = 67nm, Ly_{medium} = 35.8 nm and Lz_{medium} = 36.1 nm being the dimensions of the medium simulation cell used in Figure 4 in the manuscript (Supplementary movie 4 in the SI).

The results illustrated in Supplementary Figure 13 and Supplementary Table 4 confirm that the size of the simulation box has a major effect on the stability of the constriction node. Several simulations were performed using the small simulation cell. In particular, the initial position of the dislocation as well as the resolved shear stress acting on the dislocation glide

plane were varied. Similar to the medium configuration, the formation of the constriction node results from the competition between transmission and absorption mechanisms. In the range of stresses where both mechanisms are competing, i. e, σ_{rss_screw} = 200 – 250 MPa, the lifetime of the constriction node never exceeds 15 ps, a value 4 to 5 times shorter than the one obtained for the medium configuration (see also Supplementary movie 5). More importantly, the lifetime of the node significantly increases for the large configuration. After its formation, the constriction node rapidly migrates towards the centre of the CTB and remains in this position for at least 600 ps (see Supplementary Figure 13 and Supplementary movie 6). Note that the simulation was stopped after 600 ps because the node did not escape to the free surface. A 50% increase of Ly and Lz has therefore a considerable effect on the stability the node. The dimensions of the TEM specimen (1000nm×800nm×200nm) being much larger than the simulation cells, this suggests that the lifetime of the node could be long enough to be captured by TEM observations. Some additional effects such as surface defects and elastic interactions with the neighbouring dislocations in the CTB might further contribute to increase the stability of the node.

Supplementary Tables

Burgers vector → / Used diff. vector ↓	$a/2\,[110]$	$a/2\,[101]$	$a/2\,[0\bar{1}1]$
220	2	1	-1
200	1	1	0

Supplementary Table 1. g.b products for Burgers vectors in the $(\bar{1}11)$ slip plane of SAS1.

Burgers vector → / Used diff. spot ↓	$a/2\,[101]$	$a/2\,[011]$	$a/2\,[1\bar{1}0]$	$a/3\,[\bar{1}\bar{1}1]$	$a/6\,[112]$	$a/6\,[2\bar{1}1]$	$a/6\,[12\bar{1}]$
020	0	1	-1	$-2/3$	$2/6 \sim 0$	$-2/6 \sim 0$	$4/6$

Supplementary Table 2. g.b products for all possible dislocations in the CTB plane with $\boldsymbol{g} = \mathbf{020}$. As a rule of thumb, in FCC crystals, dislocations become invisible when $|\mathbf{g}.\mathbf{b}| \leq \mathbf{1/3}$).

Type of dislocations → / Used diff. vector ↓	$a/2\,[110]$	$a/2\,[\bar{1}01]$	$a/2\,[011]$
220	2	-1	-1
200	1	-1	0

Supplementary Table 3. g.b products for perfect dislocations in the $(\mathbf{1}\,\bar{\mathbf{1}}\,\mathbf{1})$ slip plane.

	Life time (ps)	σ_{rss_trans} (MPa)
Small: *½ Lx, ½ Ly, ½ Lz*	10-15	240
Medium: *Lx, Ly, Lz*	60	250
Large: *2*Lx, 2*Ly, 2*Lz*	> 600	265

Supplementary Table 4. Lifetime of the constriction node and critical transmission stress as a function of the size of the simulation box. Note that the simulation was stopped at 600 ps because the node did not escape to the free surface.

Supplementary Figures

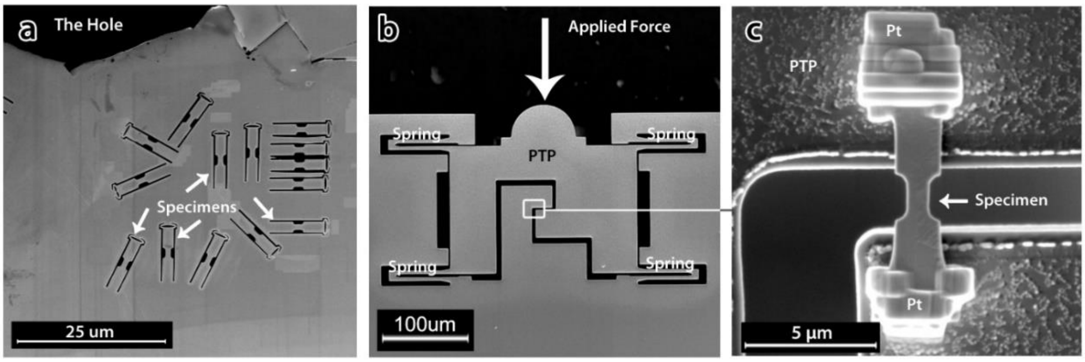

Supplementary Figure 1. Sample preparation for in-situ tensile testing. SEM images showing (a) the FIBed dog-bone specimens on the electro-polished TEM foil after the heat treatment (b) a PTP device and (c) the final configuration of the tensile specimen mounted on the PTP device.

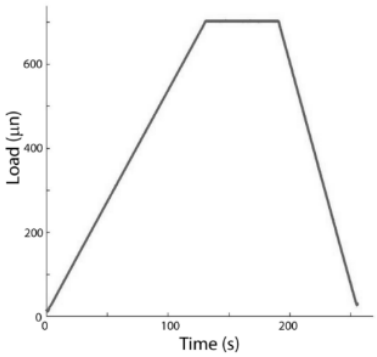

Supplementary Figure 2. Example of loading-unloading cycle. Load vs time for loading cycle 3.

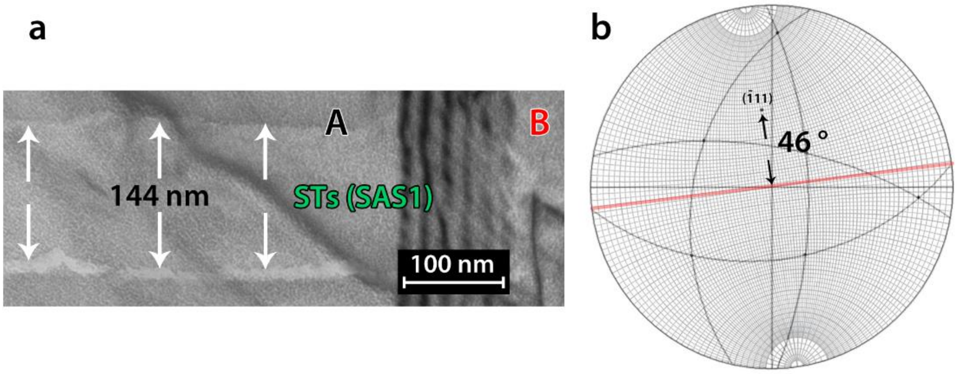

Supplementary Figure 3. Extraction of the thickness from slip traces. (a) White arrows indicate slip traces for the single arm source SAS1 activated in the $(\bar{1}11)$ slip plan in grain A. (b) stereographic projection of crystal A on a Wulff net.

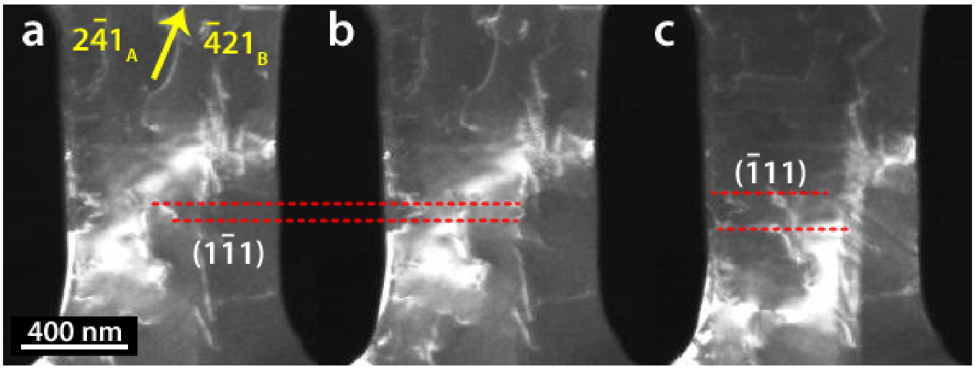

Supplementary Figure 4. Identification of the slip planes. TEM-DF snapshots from cycle 3 (a and b) with red dashed lines showing the position of the two STs induced by the activation of SAS3 in the $(1\,\bar{1}\,1)$ plane. The red dashed lines in (c) indicate the position of the STs generated by SAS2 dislocations in the $(\bar{1}\,1\,1)$ plane.

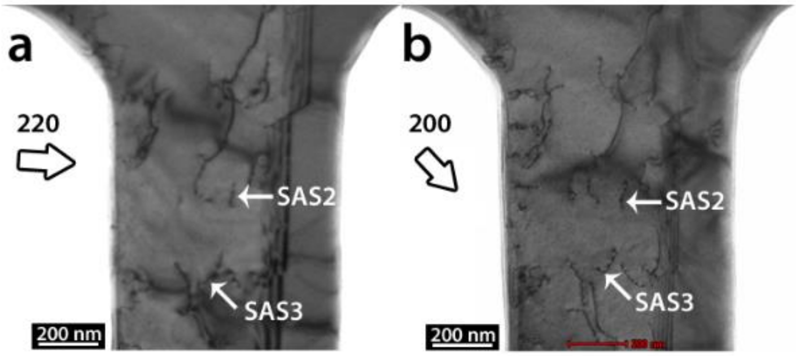

Supplementary Figure 5. Characterization of single arm sources (SASs). Two-beam TEM-BF micrographs taken with (a) g=220 and (b) g=200 diffraction vectors.

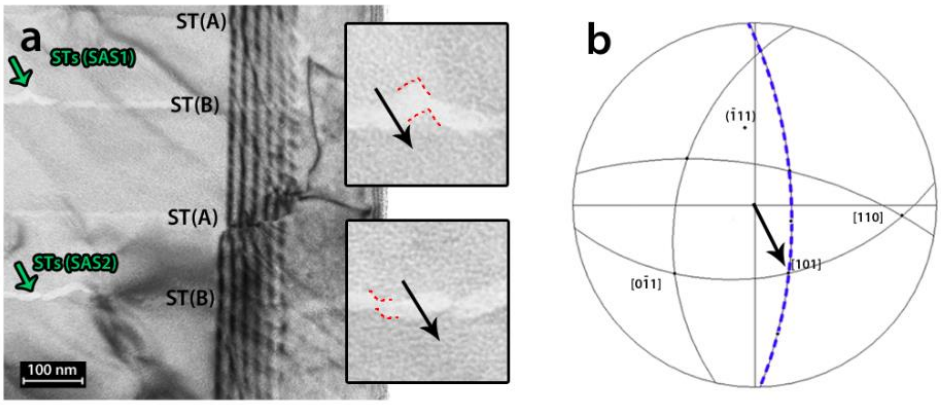

Supplementary Figure 6. Characterization of single arm sources. (a) TEM-BF micrograph showing STs from SAS1 and SAS2. The insets are magnified views of the areas marked by green arrows. (b) Stereographic projection of grain A. Dark arrows in the insets shows that the displacement of roughness features is parallel to [101] in (b).

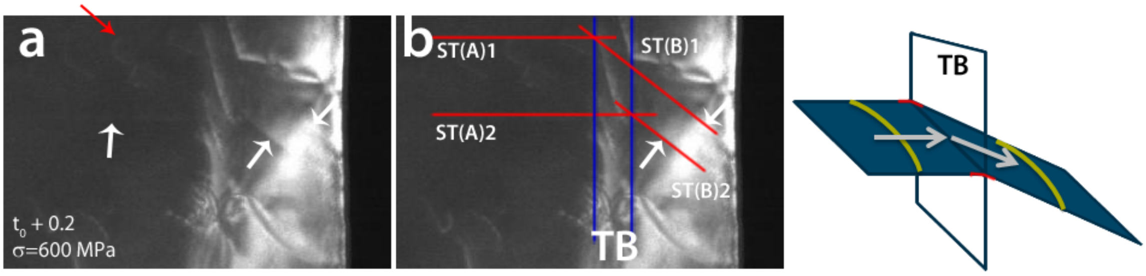

Supplementary Figure 7. Slip transmission. (a) Snapshot from the Supplementary movie 1 showing the transmission of dislocations from SAS1. White arrows indicate the position of ST(A)2, ST(B)1 and ST(B)2. The red arrow indicates the position of ST(A)1 identified by the intersection between the rotating arm of SAS1 and the surface. (b) Red and blue lines are added on the STs and the TB, respectively. It confirms that the slip traces are connected at the CTB. A schematic of transmission process is shown in (b)

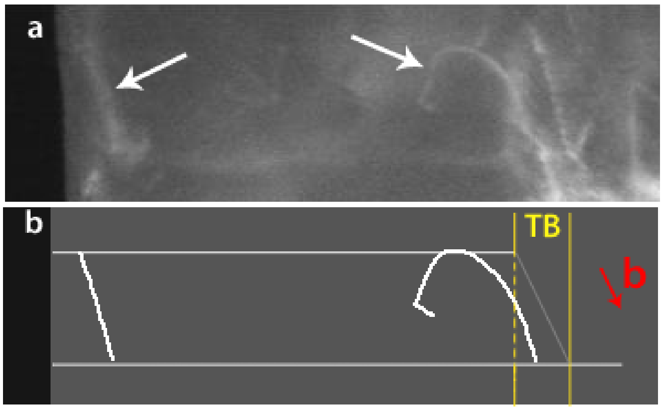

Supplementary Figure 8. Screw character of the incoming dislocations (a) Snapshot from the Supplementary movie 1 showing the operation of SAS1. White arrows indicate the SAS1 and a segment with opposite sign blocked close to the left edge of the sample (b) Schematic illustration of (a).

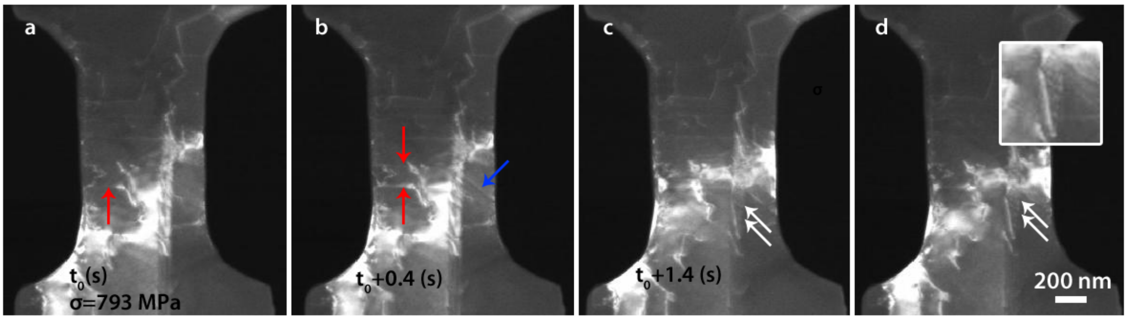

Supplementary Figure 9. Interaction between SAS2 and CTB**.** Snapshots from the Supplementary movie 2 of cycle 4 showing the operation of SAS2 (a), (b), (c) and after shutdown (d). Red and blue arrows show the freshly formed STs in grain A and grain B, respectively. White arrows indicate the dislocations absorbed at the CTB. The inset in (d) shows a magnified image of the absorbed dislocations in the CTB.

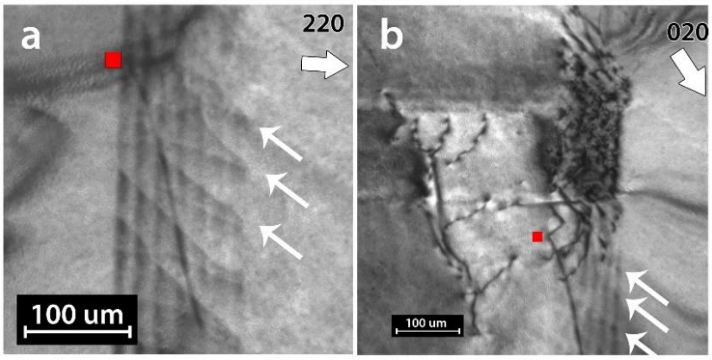

Supplementary Figure 10. Characterization of CTB dislocations. Two-beam TEM-BF images acquired with (a) 220 diffraction vector and (b) 020 diffraction vector. White arrows indicate the dislocations resulting from the absorption of screw dislocations at the CTB.

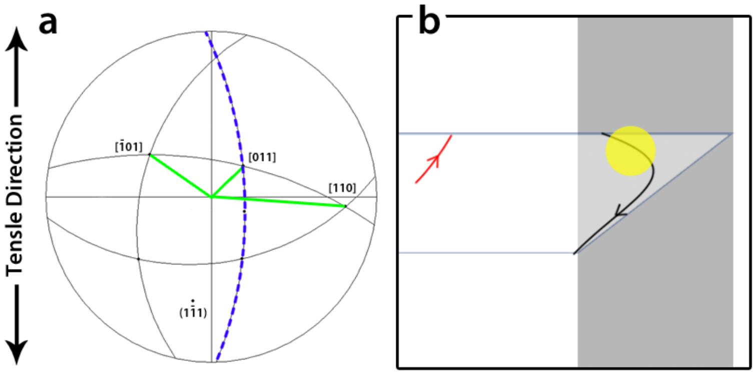

Supplementary Figure 11. Interaction of a non-screw dislocation with the CTB. (a) Stereographic projection of grain A with possible Burgers vectors on $(\mathbf{1}\,\bar{\mathbf{1}}\,\mathbf{1})$. (b) Schematic showing the configuration of the dislocation in Figure 5b in the main text. The orientation of the segment highlighted in yellow is close to $^{a}/_{2}\,[\bar{1}\,0\,1]$ Burgers vector.

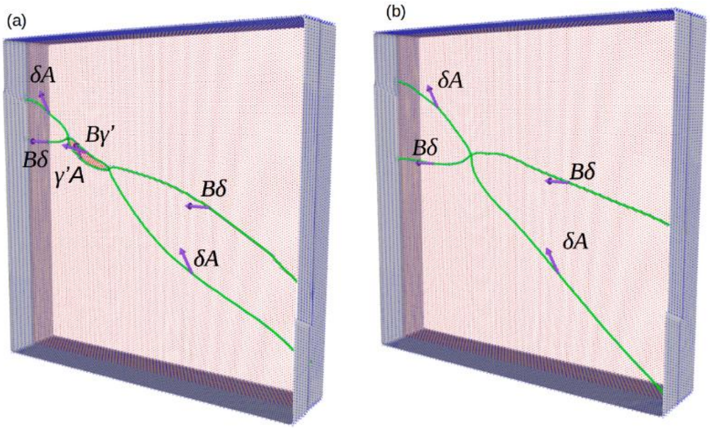

Supplementary Figure 12. Burgers vector determination of the TB dislocations upon absorption in the TB. The stress acting on the screw component is equal to 204 ± 5 MPa and below the critical stress for transmission (a) Competition between absorption on the δ CTB plane and transmission in the γ′ plane. (b) Reabsorption of the initially transmitted dislocation segment and formation of the constriction node.

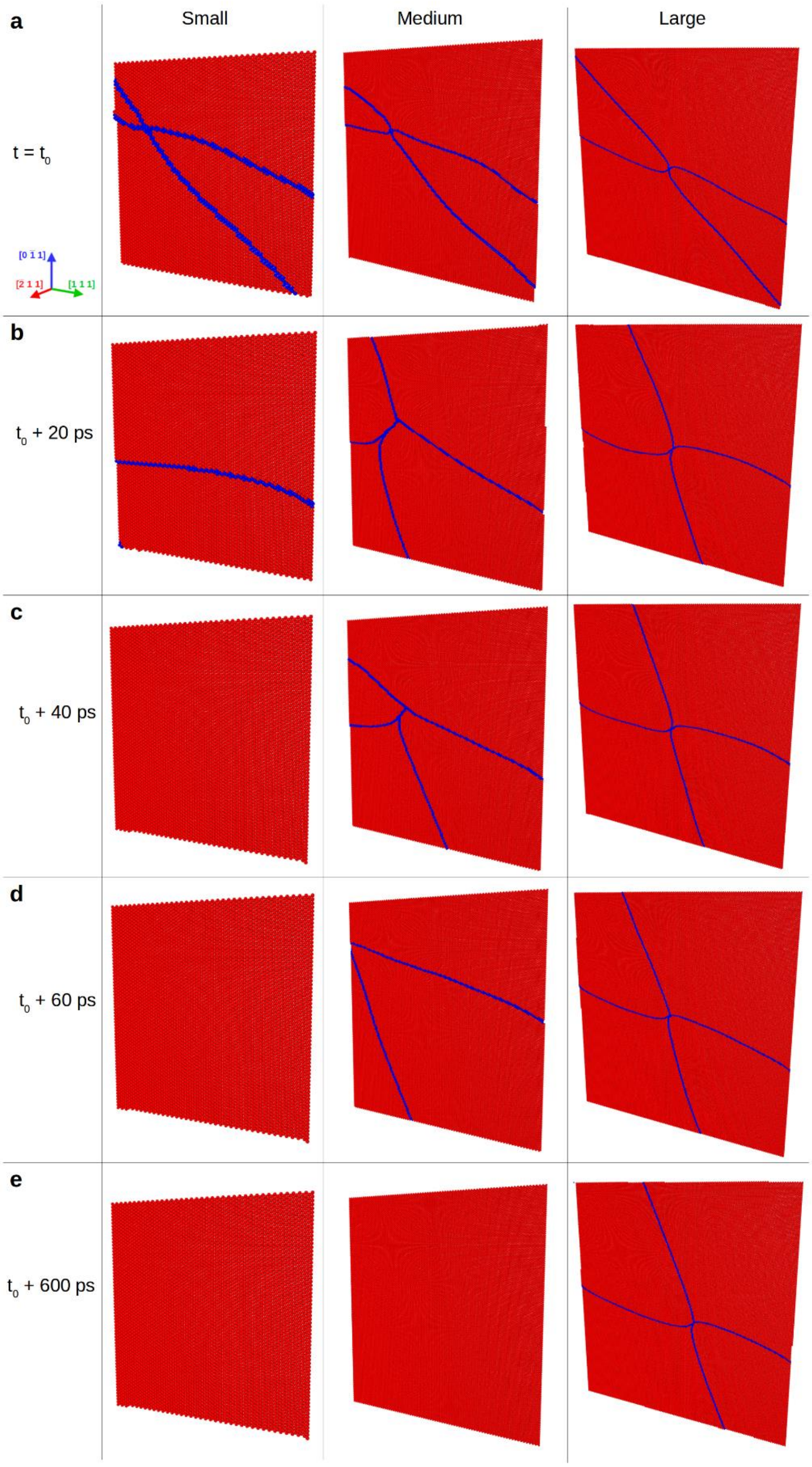

Supplementary Figure 13. Influence of the size of the simulation cell on the stability of the constriction node. (a) t_0 corresponds to the formation of the constriction node; (b) t = t_0+20 ps; (c) t = t_0+40 ps; (d) t = t_0+60 ps; e) t = t_0+600 ps.

Supplementary References

1. Kiener, D., Grosinger, W., Dehm, G. & Pippan, R. A further step towards an understanding of size-dependent crystal plasticity: In situ tension experiments of miniaturized single-crystal copper samples. *Acta Mater.* **56**, 580–592 (2008).
2. Foreman, A. J. E. The bowing of a dislocation segment. *Philos. Mag. A J. Theor. Exp. Appl. Phys.* **15**, 1011–1021 (1967).
3. Samaee, V. *et al.* Dislocation driven nanosample plasticity: new insights from quantitative in-situ TEM tensile testing. *Sci. Rep.* **8**, 12012 (2018).
4. Stukowski, A. & Albe, K. Visualization and analysis of atomistic simulation data with OVITO. *MSMSE* **18**, 015012 -2010)
