# Supplementary material for: Deciphering the interactions between single arm dislocation sources and coherent twin boundary in nickel bi-crystal
Source: Nat Commun. 2021 Feb 11;12:962. doi: 10.1038/s41467-021-21296-z (PMC7878869; doi:10.1038/s41467-021-21296-z)
Supplement: Supplementary file 2 — Description of Additional Supplementary Files [file 41467_2021_21296_MOESM2_ESM.docx]

Supplementary movie legends

File Name: Supplementary Movie 1
Description: Operation of the single arm source SAS1 with transition from transmission to absorption of the incoming curved screw dislocations.

File Name: Supplementary Movie 2
Description: Operation of the single arm source SAS2 with transition from transmission to absorption of the incoming curved screw dislocations.

File Name: Supplementary Movie 3
Description: Operation of the single arm source SAS3 with reflection of a non-screw dislocation at the CTB.

File Name: Supplementary Movie 4
Description: Medium 3D-MD simulation cell showing the interaction between curved screw dislocation and CTB. Lx_medium_ = 67nm, Ly_medium_ = 35.8 nm and Lz_medium_ = 36.1 nm.

File Name: Supplementary Movie 5
Description: Small 3D-MD simulation cell showing the interaction between curved screw dislocation and CTB. Lx_small_ = ½*Lx_medium_, Ly_small_ = ½*Ly_medium_ and Lz_small_ = ½*Lz_medium_

File Name: Supplementary Movie 6
Description: Large 3D-MD simulation cell showing the interaction between curved screw dislocation and CTB. *Lx_large_* = *Lx_medium_*, *Ly_large_* = 3/2**Ly_medium_* and *Lz_large_* = 2**Lz_medium_*
